# Mesenchymal Stem/Stromal Cell-Based Therapies in Systemic Rheumatic Disease: From Challenges to New Approaches for Overcoming Restrictions

**DOI:** 10.3390/ijms241210161

**Published:** 2023-06-15

**Authors:** Bong-Woo Lee, Seung-Ki Kwok

**Affiliations:** Division of Rheumatology, Department of Internal Medicine, Seoul St. Mary’s Hospital, College of Medicine, The Catholic University of Korea, Seoul 06591, Republic of Korea; tdomu1029@gmail.com

**Keywords:** systemic rheumatic diseases, mesenchymal stem/stromal cells, rheumatoid arthritis, systemic lupus erythematosus, systemic sclerosis

## Abstract

Systemic rheumatic diseases, such as rheumatoid arthritis, systemic lupus erythematosus, and systemic sclerosis, are chronic autoimmune diseases affecting multiple organs and tissues. Despite recent advances in treatment, patients still experience significant morbidity and disability. Mesenchymal stem/stromal cell (MSC)-based therapy is promising for treating systemic rheumatic diseases due to the regenerative and immunomodulatory properties of MSCs. However, several challenges need to be overcome to use MSCs in clinical practice effectively. These challenges include MSC sourcing, characterization, standardization, safety, and efficacy issues. In this review, we provide an overview of the current state of MSC-based therapies in systemic rheumatic diseases, highlighting the challenges and limitations associated with their use. We also discuss emerging strategies and novel approaches that can help overcome the limitations. Finally, we provide insights into the future directions of MSC-based therapies for systemic rheumatic diseases and their potential clinical applications.

## 1. Introduction

Systemic rheumatic diseases such as rheumatoid arthritis (RA), systemic lupus erythematosus (SLE), and systemic sclerosis (SSc) are inflammatory conditions associated with immune system dysregulation. RA is a chronic systemic disease primarily affecting the musculoskeletal system, including joints, tendons, and other connective tissues, significantly reducing the quality of life of those affected [1]. Significant advancements have been achieved in the field of targeted therapies for RA over the last decade. In recent times, there has been a notable expansion in the range of treatment options for RA. This includes biological or targeted synthetic disease-modifying antirheumatic drugs (b/tsDMARDs) such as anti-TNF-α blockers, abatacept, tocilizumab, and Janus kinase inhibitors. These therapies have demonstrated remarkable effectiveness in managing the symptoms and progression of RA. However, despite the advancements, the effectiveness of these therapies may still be suboptimal in certain patients. Additionally, the use of these therapies can be associated with significant risks, such as an increased susceptibility to infections and malignancies. As a result, there is an ongoing need to explore and develop novel treatment alternatives for RA to address these limitations and provide better outcomes for patients [2]. SLE is an autoimmune disorder characterized by auto-antibodies targeting nuclear and cytoplasmic antigens. This disorder causes inflammation in multiple systems and has diverse clinical symptoms, with an unpredictable disease course involving flare-ups and improvement [3]. At present, the primary treatment approaches for SLE involve the use of corticosteroids and immunosuppressants. However, long-term remission is achieved by only a few patients. Importantly, immunosuppressive therapies often fail to prevent disease relapse in more than half of the patients, and high-dose treatments can even increase the risk of severe infections and mortality. Given the unmet medical needs associated with SLE, which include inadequate disease control, diminished health-related quality of life, comorbidities, the toxicity of most therapies, and reduced survival, there is a critical need for new therapeutic agents to address these challenges [4]. SSc is a rheumatic disease where the body’s immune system inadvertently damages small blood vessels, causing widespread fibrosis in the skin and internal organs. This condition can result in various clinical symptoms, ranging from minor issues such as Raynaud’s phenomenon and fatigue to more severe complications, including pulmonary arterial hypertension and lung fibrosis [5]. An important factor contributing to poor prognosis in patients with SSc is the abnormal accumulation of collagen in the skin and various organs, including the lungs, heart, and kidneys. Unfortunately, fibrosis, characterized by this excessive collagen deposition, is typically irreversible. Currently, there is a lack of available treatment options for SSc that are both safe and effective in managing fibrosis in the affected organs. Systemic rheumatic diseases have complex pathophysiology and diverse symptoms. Therefore, conventional treatments may not be effective for cases with poor prognoses, leading to irreversible disability and organ damage. Cellular therapies have been developed with the aim of restoring immunologic self-tolerance, with the goal of achieving long-lasting remissions or promoting tissue regeneration. The recently investigated mesenchymal stem/stromal cell (MSC)-based therapy is promising in treating systemic rheumatic disease. MSCs are easily isolated and cultured in vitro, and their immune privilege allows for transferring allogeneic cells to patients.

In this review, we have provided a comprehensive overview of MSCs, including their definitions and immune-regulatory mechanisms. Our focus has been on recent preclinical and clinical studies on systemic rheumatic diseases, such as RA, SLE, and SSc. However, despite encouraging preclinical outcomes in various animal disease models, most registered clinical trials using MSC-based therapy for systemic rheumatic diseases have not met expectations. This can be attributed to the disadvantages of MSCs, such as heterogeneity, immunogenicity, and low survival rates. Therefore, we highlight new strategies to overcome these challenges and provide insights into the next generation of MSC-based therapies for systemic rheumatic diseases.

## 2. Characteristics of MSCs

MSCs were first isolated from the bone marrow (BM) in 1968 by Friedenstein [6]. They are a diverse group of fibroblast-like precursor cells not involved in blood cell formation. In addition, MSCs can differentiate into multiple cell types, including osteoblasts, adipocytes, and chondrocytes. They possess distinctive biological properties, including their ability to self-regenerate over extended periods, produce biologically active substances, exhibit antimicrobial properties, modulate immune responses, and actively locate and move into areas of damaged tissue [7]. MSCs possess a great capacity for healing autoimmune and inflammatory conditions as they can regulate the characteristics and functional qualities of immune cells. Through decades of research, MSCs are currently isolated from various organs in the human body, including the BM, adipose tissue, synovial fluid, cartilage, skin, peripheral blood, umbilical cord blood, liver, lung, and spleen. MSCs are also relatively easy to culture and have excellent proliferation capacity, enabling them to proliferate many cells [8]. Therefore, MSCs are currently utilized in many research fields, focusing on their various characteristics and functions.

In the past, there has been a tendency to use the terms “mesenchymal stromal cell” and “mesenchymal stem cell” interchangeably. However, ongoing discussions and evolving research have led to a better understanding of their specific definitions and distinguishing characteristics. Historically, the term “mesenchymal stem cell” was employed to describe cells that possessed the capacity for self-renewal and differentiation into various types of cells. However, more recent studies have suggested that the term “stem cell” might be oversimplified and potentially misleading when applied to these cells. To address the confusion, some experts proposed the use of a more precise term, “mesenchymal stromal cell,” to describe cells that display mesenchymal characteristics and possess therapeutic properties. This terminology aims to provide a clearer and more accurate description of these cells. The transition from using the term “mesenchymal stem cells” to “mesenchymal stromal cells” signifies the growing understanding that the main therapeutic mechanism of these cells might not be their ability to differentiate into various cell types. Instead, it emphasizes their role in paracrine signaling and immunomodulation, which are considered the primary therapeutic effects of these cells. This shift in terminology reflects the evolving knowledge about their therapeutic mechanisms [9].

Cell therapy with MSCs has a great application value in systemic rheumatic disease. They express low HLA-II levels, implying their possible use in autologous and allogeneic methods [10]. The interest surrounding the MSCs field was initially based on their tissue and organ regeneration and self-renewal capacity. Subsequently, given their immunomodulatory properties, MSC-based therapies have broadened their therapeutic use to chronic inflammatory diseases. MSCs have attracted much attention owing to their ability to regulate the immune system, making them useful in allogenic applications [8]. Newer studies have revealed that paracrine factors, mitochondria transfer, and extracellular vesicle secretion can influence the impact of MSCs [8,11]. MSCs release growth factors, chemokines, cytokines, and miRNAs, which act on neighboring cells and tissues. These substances can promote healing and restore a healthy environment in damaged tissues. MSC transplantation or administering isolated secreted factors can facilitate the delivery of these substances to the injured tissues [12]. Due to these benefits, clinician scientists have conducted numerous research to assess the efficacy and safety of MSCs.

## 3. Immunomodulatory Properties of MSCs

MSCs possess immunomodulatory functions that effectively regulate the immune response. The effects of MSCs on T cells have been extensively studied, and they have been found to modulate T cell responses through various mechanisms. MSCs inhibit the proliferation and activation of T cells, induce the generation of regulatory T cells (Tregs) that suppress immune responses, and promote the differentiation of naïve T cells into T helper 2 (Th2) cells that further suppress immune responses [13,14]. Additionally, MSCs reduce the production of pro-inflammatory cytokines, such as interferon (IFN)-γ and tumor necrosis factor α (TNF-α), by T cells and enhance the production of anti-inflammatory cytokines, such as interleukin (IL)-10 [15]. The effects of MSCs on B cells are less well-characterized than that on T cells. However, studies suggest that MSCs can suppress B cell proliferation and differentiation, inhibit the production of immunoglobulins, and reduce their activation and survival. Moreover, MSCs can indirectly affect B cells by modulating the function of other immune cells, such as T cells and dendritic cells [16,17]. MSCs also modulate macrophage and dendritic cell function by reducing pro-inflammatory and increasing anti-inflammatory cytokine production, promoting the differentiation of anti-inflammatory macrophages and regulatory dendritic cells, and inhibiting the phagocytic activity of macrophages [18]. In summary, the ability of MSCs to modulate various immune cells makes them promising for treating immune-related disorders. Their potential for targeted immunomodulation is an exciting research field with numerous potential applications for various diseases.

## 4. Rheumatoid Arthritis

Rheumatoid arthritis (RA) is the most prevalent inflammatory rheumatic disease, identified by synovitis involving multiple joints, destructive articular change, and extra-articular complications. RA is characterized by inflammation caused by cytokines and inflammatory cells. Treatments, including biological disease-modifying anti-rheumatic drugs (DMARDs), such as monoclonal antibodies that target TNF-α and IL-6, and Janus kinase inhibitors, are highly effective in managing RA symptoms in clinical settings [1]. Despite treatment with multiple drugs, only a small percentage of patients can achieve lasting remission from the disease. Most patients experience frequent relapses and brief periods of disease remission. Consequently, innovative and successful treatment methods are required, specifically for RA, which is challenging to manage. MSCs can offer a comprehensive solution for all the challenges of treating RA. The ultimate objective for treating RA is to recover immune tolerance. MSCs are gaining substantial attention owing to their anti-inflammatory, paracrine, and reparative properties [7].

### 4.1. MSC-Based Therapy and Mode of Action in RA

MSCs have gained attention as a potential therapy for RA due to their immunomodulatory properties. In vitro studies have played an essential role in understanding the mechanisms underlying the immunomodulatory properties of MSCs and their potential for use in RA treatment. These studies have revealed that MSCs can inhibit immune cell activation, which is involved in RA’s pathogenesis. An investigation concerning the effects of MSCs on dendritic cells, which play a role in RA’s pathogenesis by activating T cells, revealed that MSCs could inhibit the differentiation and maturation of dendritic cells. Additionally, MSCs could reduce the dendritic cells’ ability to activate T cells [19]. In another study, the proliferation and differentiation of B cells producing auto-antibodies associated with RA could be inhibited by MSCs [20].

Furthermore, Usha et al. co-cultured human adipose-derived MSCs with peripheral blood mononuclear cells (PBMCs) from patients with RA [15]. The results revealed that MSCs increased the proportion of Tregs and CD4+CD25+FoxP3 levels. MSCs can also inhibit the production of pro-inflammatory cytokines such as TNF-α, IL-1β, and IL-6—key RA inflammation drivers [21].

Recent preclinical studies have evaluated the safety and efficacy of MSCs in RA animal models, including mice with collagen-induced arthritis (CIA). In RA, a breakdown in the body’s ability to distinguish body tissues from foreign bodies activates T cells that attack the joints, resulting in long-term inflammation. The involvement of MSCs in modulating T cell activity, which includes inhibiting T cell proliferation and inducing the activation of Tregs, has been suggested [22]. A study uncovered that human umbilical cord-derived MSCs (UC-MSCs) reduced inflammation and joint destruction in a RA mouse model. Human adipose tissue-derived MSCs (AT-MSCs) increased the proportion of Th17 cells expressing IL-10 in the CIA mouse model, consistent with other in vitro studies [23]. Intravenous (IV) infusion of human UC-MSCs decreased the number of T follicular helper (Tfh) cells in the spleen of mice with CIA and suppressed Tfh’s ability to induce B lymphocyte differentiation [24]. The ability of MSCs to prevent B cells from properly functioning relies on their interaction with T cells. This means that the impact of MSCs on Tfh cells could be responsible for the indirect suppression of B cells. Additionally, AT-MSCs prevented bone loss in mice with CIA by stopping osteoclast formation in response to RANKL and reducing the precursors that become osteoclasts in the BM [25]. When BM-derived MSC (BM-MSC) therapy was administered to animals with induced RA, a considerable decrease in inflammation, joint swelling, and cartilage damage was observed compared to the untreated group [26]. Generally, based on preclinical research, MSCs may be able to treat RA effectively owing to their immunomodulatory and tissue regeneration characteristics.

### 4.2. MSC-Based Therapy and Recent Clinical Applications in RA

According to recent clinical trials, stem cell therapy may be effective for patients with RA (Table 1). A phase I/IIa clinical trial revealed that IA autologous BM-MSCs injection could safely and effectively reduce joint pain and swelling and improve joint function in patients with RA [27]. As a treatment of systemic infusion, successful results of clinical trial on the effects of IV allogenic UC-MSCs in active RA patients were reported [28]. Similarly, Ghoryani et al. published successful clinical trial results on the effects of IV autologous BM-MSCs in patients with refractory RA [29]. The results indicated a notable reduction in the number of Th17 cells, disease activity score 28-erythrocyte sedimentation rate (DAS28-ESR), and visual analogue scale (VAS) after 12 months of MSC-based therapy for patients with refractory RA. Another clinical trial to evaluate the safety and efficacy of IV allogeneic human UC-MSCs in patients with active RA revealed long-term beneficial effects [30]. The results revealed that the therapy was safe and well-tolerated, significantly improving disease activity, joint function, and quality of life for 3 years. The clinical trials in this section demonstrate that autologous and allogeneic MSC transplantation is safe and effective for patients with refractory RA. However, additional studies are required to determine the cell dosage, administration route, and treatment timing for optimal efficacy and long-term safety.

## 5. Systemic Lupus Erythematosus

Systemic lupus erythematosus (SLE) is a long-lasting autoimmune disease that affects the entire body, leading to organ dysfunction and significantly affecting the patient’s quality of life [3]. The condition is characterized by alternating periods of remission and relapse. Unfortunately, no definitive cure or universally effective treatment exists for SLE. Recent treatments such as antimalarial, immunosuppressant, and glucocorticoid drugs are available; however, they often result in adverse patient reactions. Moreover, many patients with lupus still do not respond adequately to existing therapies [38]. Therefore, cellular therapies, specifically MSCs, are areas of growing interest due to their potential therapeutic benefits for SLE.

### 5.1. MSC-Based Therapy and Mode of Action in SLE

SLE’s etiology is not well comprehended. However, dysregulation of various immune cells and cytokines is involved in its pathogenesis. In SLE, plasmacytoid dendritic cells (pDC) stimulate the immature B-cells into plasma cells [39]. When B cells become excessively activated and produce abundant autoantibodies, it results in the inflammation of multiple organs such as the skin, joints, and internal organs such as the kidneys [40].

According to several studies, MSCs can control SLE’s activity by impeding B cell differentiation and proliferation. In the lupus nephritis mouse model, human gingival-derived MSCs improved proteinuria and histopathological scores of nephritis by inhibiting B cell activity via the CD39-CD73 pathway in vitro and vivo [41]. Additionally, MSCs could block the differentiation of B cells into plasma cells via the PD-1/PD ligand pathway [42]. Administering MSCs in a murine lupus model demonstrated an ability to stimulate the expansion of IL-10-producing regulatory B cells (Bregs) and inhibit overactive inflammatory reactions [43]. In patients with SLE, injecting human-umbilical-cord-derived MSCs reduced Th17 cells in the bloodstream and increased Treg cells [44]. Furthermore, injecting human BM-MSCs inhibited glomerulonephritis, lowered autoantibodies production, decreased proteinuria, and enhanced survival rates in NZM/W F1 mice. This was achieved by inhibiting Tfh cell development [45]. MSCs can reduce SLE’s activity by inhibiting pDC maturation associated with IFN-α production. In adriamycin (ADR)-induced nephropathy mice, human BM-MSCs inhibit IFN-α expression and suppress kidney inflammation [46].

### 5.2. MSC-Based Therapy and Recent Clinical Applications in SLE

Several clinical trials of MSCs in SLE have been completed (Table 2). Clinical benefit was not observed in the first study using autologous MSCs in patients with SLE [47]. However, in another study, 15 patients with active and refractory SLE were treated with allogeneic MSC transplantation, and all the patients achieved disease remission. Within one year, a significant decrease in SLE disease activity score (SLEDAI score), anti-dsDNA level, and 24 h proteinuria level was observed [48]. Two clinical studies evaluated the results of the IV injection of allogeneic BM-MSCs and UC-MSCs in patients with refractory SLE. They demonstrated that the allogenic MSCs substantially improved disease remission and renal function and reduced autoantibodies in patients with severe lupus and limited response to conventional therapy [49,50]. A study published in 2022 reported the results of a phase I clinical trial investigating the safety and feasibility of using a single dose of allogeneic AT-MSCs infusion to treat refractory lupus nephritis [51]. The study observed that the treatment was safe and well-tolerated. However, the most significant beneficial effect of proteinuria and SLE was seen after one month and six months, respectively. Therefore, a single AD-MSC dose may be insufficient to keep refractory LN in long-term remission.

Most clinical studies have demonstrated that MSC therapy for SLE is effective but with varying degrees of success. The variability in the effectiveness of these MSC therapies may be attributed to factors such as the quantity and kind of MSCs used, the health status of patients before treatment, and concurrently using other immunosuppressants. Most SLE clinical trials involved a single MSC dose, with inconsistent outcomes. Therefore, larger studies are required to establish the optimal dosage that produces favorable results.

## 6. Systemic Sclerosis

Systemic sclerosis (SSc) is a systemic connective tissue disease caused by an immune system malfunction. This condition is characterized by blood vessel damage, immune system abnormalities, and fibrosis on the skin and internal organs [5]. SSc significantly affects various body parts, including the lungs, heart, kidneys, digestive system, and musculoskeletal system. It also considerably influences the life span, quality of life, and mortality [59]. Studies have revealed that over 90% of individuals with SSc exhibit signs of interstitial lung disease (ILD) on autopsy, as lung function changes indicate ILD in 40%–75% of patients with SSc. ILD is characterized by chronic inflammation and fibrosis that progress to respiratory failure and death [60,61]. SSc is currently deemed untreatable. Developing immune-modifying drugs and antifibrotic treatments has increased their accuracy and effectiveness. Nevertheless, these remedies delay the disease’s progression and rarely reverse its symptoms. Additionally, maintenance therapies have potential side effects, such as infections, and may increase the possibility of comorbidities over time [5]. MSCs have been explored in various medical fields due to their ability to modify the immune response, support cell growth, and promote blood vessel formation. They can also combat some of the processes that contribute to SSc progression.

### 6.1. MSC-Based Therapy and Mode of Action in SSc

Studies indicate that a combination of genetic predisposition and environmental factors can lead to SSc onset, damaging the endothelial cells, injuring small blood vessels, activating the immune system, and causing tissue fibrosis [5]. Cytokines released by activated B and T cells cause endothelial cells to transform into myofibroblasts. Fibrosis occurs when myofibroblasts produce excessive collagen and other extracellular matrix proteins [62]. MSCs may be promising candidates for treating SSc, considering their angiogenic properties and immunomodulatory role [63].

Studies have used MSCs in bleomycin (BLM)-induced animal SSc models. In the first mouse study exposed to BLM, lung fibrosis and inflammation reduced after murine MSC administration [64]. Similarly, human UC-MSCs displayed antifibrotic properties and lung repair function via reduced transforming growth factor β (TGF-β) and IFN-γ [65]. Afterward, skin fibrosis improved following the introduction of UC-MSCs in an SSc model induced by BLM. This was accompanied by Th-17 cell inhibition and reduced collagen production [66]. In another animal model—a hypochlorite (HOCl)-SSc model—intravenous BM-MSC injection demonstrated therapeutic effects, including reduced skin and lung fibrosis, inflammation, and anti-Scl-70 autoantibodies [67]. UC-MSCs use in the HOCl-SSc model also prevented fibrosis [68]. Overall, preclinical studies conducted in SSc animal models have revealed the potential of MSCs in the involvement of the skin and lungs in SSc.

### 6.2. MSC-Based Therapy and Recent Clinical Applications in SSc

Researchers have recently studied MSC use in treating patients with SSc (Table 3). An open phase I clinical trial reported the safety and potential efficacy of the autologous stromal vascular fraction (SVF) injection in the hands of patients with SSc. After six months, significant improvement in hand symptoms, including pain, edema, and Raynaud’s phenomenon, was observed [69]. The same result was demonstrated in a study with a long-term follow-up of 22–30 months [70]. Therefore, subcutaneous SVF injection was identified as a potentially effective SSc therapy. However, in a recent double-blind, multicenter phase II trial, no significant difference in hand function existed between the AD-SVF injection (n = 20) and placebo (n = 20) groups [71]. Therefore, studies should be conducted with larger populations presenting with homogenous SSc phenotypes to evaluate the advantages of AD-SVF injection precisely.

In the first study of a patient who received IV BM-MSC infusion in treating progressive diffuse SSc, the number of painful ulcerations decreased [72]. A study was published on IV BM-MSC infusion for 20 patients with SSc. The results indicated its safety, and 15 of the 20 patients experienced improvements in skin thickening [73]. A clinical trial investigated the potential benefits of combined plasmapheresis and allogeneic MSC-based therapy for SSc. The results revealed significant improvements in skin thickness, lung function, and quality of life at six months post-treatment, which were sustained for up to 18 months [74]. In addition, the therapy was well-tolerated, and no severe adverse events were reported.

Phase I and II studies have investigated the use of MSCs, locally and systemically, and have reported their general safety and potential effectiveness in stabilizing or improving SSc. Systemic MSC administration may apply to diffuse cutaneous thickening and internal organ fibrosis, such as interstitial lung disease. On the other hand, local MSC-based therapy has been used to treat digital ulcers and skin fibrosis, particularly perioral, improving vascularization and elasticity. These methods could be adjunct treatments alongside standard drug treatments for refractory disease. However, further studies are needed to evaluate the long-term effects and persistence of MSC engraftment or infusion in patients with SSc.

**Table 3:** Major studies including clinical applications of MSCs in SSc patients.

Published Year	MSCs Source	Sample Size and Target	Administration Routes	Outcome	Serious Adverse Events	Ref
2011	Allogenic BM	5, Diffuse type SSc	IV, single, 1 × 10^6^ cells/kg	Slight improvement of MRSS.	-	[75]
2017	SVF	12, SSc, Hand dysfunction	SC	Improvement of hand pain, finger edema, and Raynaud’s phenomenon	-	[70]
2017	Autologous AT with PRP	7, SSc, Oro-facial fibrosis	SC	Improvement of perioral fibrosis.	-	[76]
2017	Allogenic UC	14, Diffuse type SSc (3 with ILD)	IV, single, 1 × 10^6^ cells/kg (Combined with plasma exchange)	Improvement of MRSS and ILD. ↓: anti-Scl70 antibody, TGF-β, VEGF	-	[74]
2019	Autologous AT	62, SSc, Oro-facial fibrosis	SC	Improvement of perioral fibrosis.	1 case of wound infection	[77]
2019	Autologous AT	38 SSc, Digital ulcer (25 Treatment vs. 13 Placebo)	SC	Improvement of ischemic digital ulcers in all of treatment group. ↓: Pain ↑: Finger capillary.	-	[78]
2020	SVF	18, SSc, Hand dysfunction	SC, single, 3.61 × 10^6^ cells (average)	Improvement of skin fibrosis, hand edema, and active ulcers.	-	[79]
2022	SVF	40 SSc, Hand dysfunction (20 Treatment vs. 20 Placebo)	SC	No difference between two groups.	-	[71]
2022	Autologous AT	88 SSc, Hand dysfunction (48 Treatment vs. 40 Placebo)	SC	No difference between two groups.	1 case of aspiration pneumonia in treatment group	[80]

UC: umbilical cord; BM: bone marrow; AT: adipose tissue; SVF: stromal vascular fraction; PRP: platelet-rich plasma; SC: subcutaneous injection; IV: intravenous; MRSS: modified Rodnan skin thickness score; ILD: interstitial lung disease.

## 7. MSC-Based Therapy: Current Challenges and Limitations

The clinical studies mentioned above have validated the possibility and safety of MSCs for systemic rheumatic diseases. The efficacy of MSCs in treating systemic rheumatic disease has varied in clinical trials. Some studies have observed promising results, while others have reported no significant improvement in disease outcomes. Various obstacles contribute to the failure of MSC clinical development, which must be resolved to optimize its curative potential.

MSCs exist in limited quantities in adult tissues. MSCs can hypothetically be obtained from almost any tissue within the human body; however, their main sources are BM and adipose tissue. In choosing a suitable source of cells, the healthcare provider considers the disadvantages of obtaining the samples and the possible adverse events on the donor when collecting the cells. For instance, the invasive procedure for collecting BM-MSCs may cause bleeding, pain, and infection [81]. Therefore, new attempts have been made to discover alternative MSC sources, and nasal turbinate is recognized as a potential substitute in the medical field [82].

Generally, the original MSCs are thought to have low immunogenicity [10]. Most MSC products are produced by multiplying a few cells from donors, which may elevate the immunogenicity of MSCs due to inappropriate manufacturing [83]. In a study of differentiated MSCs, MHC-I and MHC-II expression increased their immunogenicity during differentiation [84]. According to another study, repeated intra-articular injections of allogeneic MSCs most likely result in unfavorable immunogenicity compared to using autologous cells under the same culture conditions [85].

Regarding the donor, autologous MSCs are readily available and do not trigger immune rejection, unlike allogenic MSCs. However, the MSCs obtained from patients with SLE have decreased migratory and proliferative ability. Therefore, autologous MSCs may not provide considerable benefits compared to allogeneic MSCs obtained from healthy individuals [86]. The expression of several genes associated with senescence and the inflammatory microenvironment of patients with SLE alter the immunomodulatory capacities of MSCs from those patients [87]. Allogeneic MSCs are promising candidates for treating SLE. In contrast, autologous MSCs may be unsuitable for treating patients with SLE due to their defective immunomodulatory function. Heathy or young MSCs play an anti-inflammatory role, but when they undergo senescence, their role shifts to promoting inflammation caused by senescence-associated secretory phenotype (SASP) proteins. Data from in vitro study of senescent BM-MSCs induced by radiation showed the upregulation of IL-6 accompanied by undermined immunosuppressive function [88]. Moreover, senescent MSCs secrete reactive oxygen species (ROS), which results in harmful oxidative stress to the microenvironment, and this oxidative stress translates the senescent characteristics to neighboring cells [89]. In addition, the condition of inflammation in older individuals, known as inflammaging, has negative effects, and it may potentially trigger transplanted MSCs to produce increased levels of SASP, subsequently leading to a suppression of their regenerative and immunomodulatory capabilities [90]. Further research aimed at reversing the senescence and microenvironment of MSCs in patients with systemic rheumatic diseases may help autologous MSC become an effective therapy.

The heterogeneity of the cell population is a major challenge in the clinical use of MSCs. Many factors also contribute to the heterogeneity and variability of MSCs. The donor factors, including age, sex, health status, and genetic factors, can influence the quality and therapeutic potential of the MSCs [91]. Additionally, the tissue source of the MSCs can impact the cells’ characteristics. Moreover, various cell isolation techniques can result in different levels of purity and sub-populations [92]. For instance, MSCs derived from the umbilical cord exhibit comparable characteristics and functions to those from BM. However, they have lower immunogenicity and greater proliferation and differentiation efficacy than BM-MSCs [93]. Furthermore, the cell culture environment and storage conditions can also impact MSC expansion and state, leading to further heterogeneity [94]. More studies are required to identify these diverse subpopulations based on biomarkers and biological functions.

Via a continuous long-term culture, MSCs can gradually lose stem cell function and life span. This senescence reduces the proliferation and differentiation abilities of MSCs, and they cannot be expected to be effective for systemic rheumatic diseases [95]. In a study on proper cell culture methods, long-term cultures with low-density MSCs displayed a higher expansion ability and life span [96]. For clinical trials for systemic rheumatic diseases to be successful, having a standardized manufacturing process with developed technology for large-scale production and improvement of MSC functions is crucial.

Identifying the fate of MSCs in vivo is a significant concern for MSC-based therapy success. The effectiveness of MSC-based treatment is generally unsatisfactory in most cases due to insufficient MSC concentration at the target organ. Therefore, assessing the outcome of clinical trials with different MSC doses, injection intervals, and administration routes, including systemic administration via intravenous or local transplantation (for instance, intra-articular injection), becomes possible [97]. Expanding our comprehension of the underlying mechanisms of treatments necessitates comprehending the distribution of these cells after injection. Systemic administration is reasonable for controlling general manifestations of systemic rheumatic diseases. However, the cell function was insufficient and transient due to the low survival and migration rate of MSCs in the target tissues after transplantation [98]. Therefore, studies are ongoing for the methods to increase the migration rate of MSCs with appropriate delivery. For instance, modifying MSCs to express certain chemokine receptors may enhance migration and survival [99].

Negative effects have also been reported in MSC-based treatments. In recent years, there has been some reports documenting adverse events and side effects associated with the use of MSC-based therapy. Potential side effects of MSC-based therapy may contain various risks, such as infection resulting from viral or mycoplasma-contaminated MSCs, xeno-contamination due to the use of cell media containing animal serum, the development or progression of malignant tumors, and the occurrence of thromboembolism and major organ fibrosis. To mitigate these potential risks, it is crucial to conduct comprehensive assessments of the MSCs manufacturing process and implement rigorous monitoring protocols for patients undergoing MSC therapy. Most of these side effects could be caused by uncontrollable differentiation of MSCs or unpredictable events during cell-culture. Considering these major causes, various therapeutic strategies have been proposed. Among them, cell-free strategies, such as MSC-derived secretomes, can serve as alternatives, capable of preventing the various side effects while preserving the advantages of MSC treatment [100].

MSC-based therapy is promising as a treatment option for systemic rheumatic diseases; nonetheless, several limitations and disadvantages are associated with their use. Therefore, novel strategies are needed to address these challenges and develop standardized protocols for MSC-based therapy.

## 8. MSC-Based Therapy: Novel Approaches to Overcoming Challenges

The therapeutic effects of MSCs have been proven. In a meta-analysis conducted to evaluate the safety of MSC therapy in humans, no life-threatening adverse events were observed. Only non-serious adverse events, such as temporary fever, injection site reactions, insomnia, and constipation, were identified [101]. However, limitations to their clinical application also exist. Thus far, various strategies have been put forward to improve the efficacy of MSCs, including enhancing their immunomodulatory and regenerative roles in patients with systemic rheumatic diseases.

### 8.1. Extracellular Vesicles from MSCs

Despite being initially studied for their pluripotency, the increasing focus on MSCs is based on their paracrine impact. Studies have suggested that the actions of MSCs are primarily influenced by their secretomes, including soluble factors and extracellular vesicles (EVs). MSC-derived EVs have similar or better therapeutic efficacy for systemic rheumatic diseases [102]. Recent studies conducted in vitro and in RA animal models have discovered that EV components can be transferred into immune cells and affect their functions [103]. Moreover, when exosomes from MSCs were administered to lupus mice models, they induced M2 macrophage and Treg cells and relieved lupus nephritis [104]. Additionally, miR-196b-5p in MSC-derived exosomes significantly inhibited the collagen type 1 expression in BLM-induced skin fibrosis in mice and suppressed skin fibrosis [105].

MSC-derived EVs can be an excellent substitute for MSC therapy because they have comparable biological properties to MSCs but with less immunogenicity and greater stability [102]. Furthermore, exosome therapy may be a safer alternative to cell therapy as it has no risk of tumor formation from stem cells [106]. The extraction and purification of MSC-derived EVs are comparatively more effortless and consistent than those of the MSCs [107]. Consequently, producing therapeutic agents becomes more efficient and consistent. In addition, EVs can cross biological barriers, such as the blood–brain barrier, more smoothly than MSCs [108]. This quality suggests that EVs can treat the central nervous system involvement of autoimmune diseases. The secretome of senescent MSCs has been identified as a significant contributor to inflammaging. So, non-senescent MSC secretome could become a therapeutic strategy for cell-free therapy, not only in systemic rheumatic disease but also in age-related disease. For example, engineering the composition of MSC secretome could make MSC-based therapy able to target specifically dysfunctional cells and tissues. The utilization of secretome-based nanomedicine has the potential to expand the therapeutic options available for immune-mediated rheumatic disease and age-related disease associated with inflammaging [89].

To ensure effective therapeutic application, it is crucial to establish consistent protocols for isolating, purifying, and storing secretomes. Validation tests should also be implemented. Additionally, it is important to clearly define the appropriate dosage and administration route for secretome-based treatments [109].

In summary, EVs obtained from MSCs offer numerous advantages over the cells as a treatment option for autoimmune disorders. These benefits include simpler extraction and purification, reduced immunogenicity, superior tissue penetration, and the ability to maintain therapeutic properties.

### 8.2. Biomaterial Strategies Applied to MSC-Based Therapy

The therapeutic potential of MSCs for treatment has been demonstrated. However, challenges are associated with the targeted delivery and longevity of MSCs at the desired sites. Thus, various biomaterial-based strategies have been developed to improve the efficacy of MSCs, such as hydrogels, microspheres, nanoparticles, and scaffolds. They are beneficial as regards MSCs adherence and survival and the preservation of secreted functional components, thereby prolonging the effective duration of clinical treatment [110].

Using hydrogels or scaffolds to encapsulate MSCs is a potential strategy for enhancing the effectiveness of MSC-based therapies. This approach may increase the retention and engraftment rates of MSCs and facilitate their differentiation into specific tissue types [111]. A study successfully developed a composite of human UC-MSC-exosome and a thermosensitive hydrogel, which effectively promoted angiogenesis and wound healing in chronic diabetic wounds. The continuous release of exosomes from the composite accelerated the wound healing rate and improved epithelial regeneration. Additionally, it promoted skin appendage healing, suggesting its potential as a therapeutic approach for treating chronic diabetic wounds [112]. Tissue-engineered scaffolds can create a 3D space that imitates the natural extracellular matrix of the desired tissue, enhancing the survival and functionality of transplanted MSCs [113]. However, research on biomaterial strategies for mesenchymal stem cell therapy in systemic rheumatic diseases remains in its early stages.

### 8.3. Preconditioning/Priming and Genetic Modification of MSCs

Preconditioning of MSCs refers to pre-treating MSCs with certain molecules or environmental factors to boost their therapeutic capacity [114]. This process aims to enhance the benefit of MSCs by promoting alterations in their genetic and functional characteristics.

According to certain research, autophagy is crucial in protecting MSCs from ROS produced during oxidative stress [115]. Pre-treatment methods, such as starvation and administration of rapamycin, are commonly employed to induce autophagy in MSCs [115]. Hypoxic preconditioning enhances the immunomodulatory effects of MSCs by upregulating prostaglandin E2 and indoleamine-2,3-dioxygenase [116,117]. MSCs preconditioned with hypoxia effectively reduced pulmonary fibrosis in the BLM-induced pulmonary fibrosis model [118].

Preconditioning MSCs with pro-inflammatory mediators, such as IFN-γ, TNFα, IL-1α, and IL-1β, is frequently utilized to enhance the therapeutic efficacy of MSCs [119]. Studies have revealed that preconditioning MSCs with IFN-γ or IL-1β results in more efficient T cell proliferation inhibition, NK cell and macrophage activation, and pro-inflammatory cytokine production than untreated MSCs [120,121]. In a study using lupus-prone MRL-Fas(lpr) mice, priming MSCs with IFN-γ improved their ability to inhibit B cells and SLE progression [122].

Interestingly, the three-dimensional (3D) spheroid culture of MSCs has emerged as a promising priming method. The 3D spheroid culture can better mimic the in vivo microenvironment of MSCs—where cells interact with each other—and the extracellular matrix (ECM). This can improve cell communication and enhance the cell differentiation potential [123]. In a study using the RA mouse model, using a 3D culture method to enhance the effectiveness of UC-MSC secretomes resolved the local and systemic RA symptoms quicker than using secretomes generated using standard 2D monolayer techniques [123].

Researchers have utilized genetic engineering techniques to induce the secretion of trophic cytokines and other advantageous gene products in various preclinical models to enhance the therapeutic potential of MSCs. This strategy has been successful in animal models, with MSCs being genetically modified to produce therapeutic peptides and proteins. A study involving the CIA mouse model explored the therapeutic benefits of gene-edited amniotic MSCs that overexpress TGF-β [124]. Another study genetically modified MSCs to overexpress hepatocyte growth factor and observed that the modified MSCs ameliorated skin fibrosis [125]. These findings raise expectations for applying these technologies to systemic rheumatic diseases.

Briefly, the preconditioning/priming and genetic modification of MSCs are potentially effective for increasing their therapeutic capabilities in treating systemic rheumatic diseases. MSCs can regulate the immune system and repair damaged tissues better by modifying their genetic and functional characteristics. This modification results in better treatment outcomes for various medical conditions.

## 9. Conclusions

**Figure 1:**
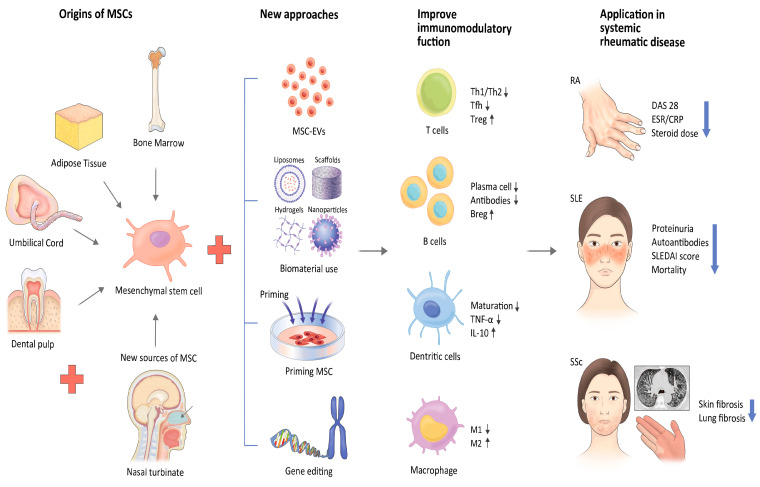
New strategies to address challenges of MSCs.

MSC-based therapies have the potential to treat systemic rheumatic diseases. However, several challenges need to be addressed. The main obstacles include heterogeneity, immunogenicity, stability, and the function of migration. Despite these challenges, new approaches, such as using genetically modified cells and EVs, are being explored to overcome limitations and improve the efficacy of these treatments. Ongoing research continues to explore the efficacy of MSC therapy for systemic rheumatic diseases. Clinical trials are being conducted to further evaluate the safety and effectiveness of MSC therapy for systemic rheumatic diseases. However, additional research is still necessary to establish its full potential and ensure its safety. In order to ensure the safety of MSC therapy, a series of tests are typically carried out. The testing procedures involve screening the donor for infectious diseases and genetic abnormalities, assessing the quality and viability of the stem cells, and verifying their identity. Before the treatment, patients may undergo a range of medical examinations and assessments to determine their suitability for the therapy. Furthermore, it is essential to closely monitor patients during and after the treatment to promptly detect any potential adverse effects and ensure the safety of the patients. Therefore, MSC-based therapies may be promising for managing systemic rheumatic diseases, providing patients with a safer and more effective alternative to traditional treatments (Figure 1). In conclusion, considering the growing understanding of the efficacy of MSCs and the development of new technologies that address existing limitations, MSC-based treatments hold significant promise as a potential solution for refractory systemic rheumatic diseases.

## Figures and Tables

**Table 1:** Major studies including clinical applications of MSCs in RA patients.

Published Year	MSCs Source	Patients	Administration Routes and Dose	Outcome	Serious Adverse Events	Ref
2013	Allogenic UC	136 (MSCs) vs. 36 (Control)	IV, single or twice, 4 × 10^7^ cells	(Only in MSCs group) ↓: CRP, RF, DAS28, HAQ TNF-α, IL-6 ↑: Tregs	-	[28]
2017	Allogenic AT	46 (MSCs) vs. 7 (Control)	IV, three-times, 1 or 2 or 4 × 10^6^ cells/kg	Just a trend for clinical efficacy in ACR20 response	1 case of lacunar infarction	[31]
2018	Autologous BM	15 (MSCs) vs. 15 (Control)	IA, single, 4 × 10^7^ cells	Improved WOMAC, VAS But, not significantly sustained beyond 12 months.	-	[27]
2018	Allogenic UC	52 (MSCs) vs. 53 (Control)	IV, single, 1 × 10^6^ cells/kg	(MSCs responder) Initial IFN-γ↑ -> IL-10↑, Treg/TH17↑	-	[32]
2019	Allogenic UC	64 (MSCs)	IV, single, 4 × 10^7^ cells	(1 year) ↓: ESR, CRP, RF, DAS28, HAQ (3 years) ↓: ESR, CRP, RF, anti-CCP, DAS28, HAQ	-	[30]
2019	Autologous BM	9 (MSCs)	IV, single, 1 × 10^6^ cells/kg	↓: DAS28, VAS ↑: Treg/TH17	-	[29]
2019	Autologous BM	13 (MSCs)	IV, single, 1 × 10^6^ cells/kg	↓: VAS, CXCL8/12/13 But, not sustained beyond 12 months.	-	[33]
2020	Allogenic UC	32 (MSCs) vs. 31 (MSCs+IFN-γ)	IV, single, 1 × 10^6^ cells/kg	(MSCs+ IFN-γ > MSCs) ↓: ESR, CRP, RF ↑: Treg/TH17	-	[34]
2020	Autologous BM	13 (MSCs)	IV, single, 1 × 10^6^ cells/kg	↑: FOXP3 expression, TGF-β1, IL-10	-	[35]
2020	Autologous BM	13 (MSCs)	IV, single, 1 × 10^6^ cells/kg	↓: CD19+ B-cell, BAFF, APRIL	-	[36]
2022	Autologous AT	15 (MSCs)	IV, single, 2 × 10^8^ cells	↓: Count of swollen/tender joint	-	[37]

UC: umbilical cord; BM: bone marrow; AT: adipose tissue; IV: intravenous; IA: intra-articular; WOMAC: Western Ontario and McMaster Universities Arthritis Index; VAS: visual analogue scale; ACR20: American College of Rheumatology 20% improvement criteria; BAFF: B cell activating factor from the tumor necrosis factor family; APRIL: a proliferation-inducing ligand.

**Table 2:** Major studies including clinical applications of MSCs in SLE patients.

Published Year	MSCs Source	Patients	Administration Routes and Dose	Outcome	Serious Adverse Events	Ref
2010	Allogenic BM	15 (MSCs)	IV, single, 1 × 10^6^ cells/kg	↓: SLEDAI scores, Proteinuria, Anti-dsDNA 2 patients relapse of proteinuria at 1 year.	-	[48]
2010	Allogenic UC	16 (MSCs)	IV, single, 1 × 10^6^ cells/kg	(8 months) Improvement of SLEDAI scores and renal function	-	[49]
2013	Allogenic BM or UC	87 (MSCs)	IV, single, 1 × 10^6^ cells/kg	(At 4 years) Remission rate: 50% Relapse rate: 23%	-	[52]
2014	Allogenic UC	40 (MSCs)	IV, twice, 1 × 10^6^ cells/kg	(12 months) ↓: SLEDAI scores, BILAG index, serum Cr, BUN, Anti-dsDNA ↑: Serum albumin and C3	-	[53]
2014	Allogenic BM or UC	81 (MSCs)	IV, single, 1 × 10^6^ cells/kg	(At 1 year) Remission rate: 60.5% Relapse rate: 22.4% ↓: SLEDAI scores, BILAG index	-	[54]
2017	Allogenic UC	9 (MSCs)	IV, twice, 1 × 10^6^ cells/kg	(At 6 years) Long-term good safety.	-	[55]
2017	Allogenic UC	12 (MSCs) vs. 6 (Placebo)	IV, twice, 2 × 10^8^ cells	No positive effect.	1 case of pneumonia	[56]
2018	Allogenic BM	3 (MSCs)	IV, single, 1.5 × 10^6^ cells/kg	↓: SLEDAI scores, Proteinuria	-	[50]
2022	Allogenic AT	9 (MSCs)	IV, single, 2 × 10^6^ cells/kg	(At 3 months) Complete response: 33.3% Partial response: 44.4% (At 6 months) ↓: SLEDAI scores (Slightly increased at 12 months)	-	[51]
2022	Allogenic BM	6 (MSCs)	IV, twice, 2~3 × 10^6^ cells/kg	Maximum tolerate dose: 3 × 10^6^ cells/kg	-	[57]
2022	Allogenic UC	6 (MSCs)	IV, single, 1 × 10^6^ cells/kg	(At 1 year) SRI-4: 83.3% ↓: CD27IgD double negative B cells, Anti-dsDNA ↑: GARP-TGFβ	-	[58]

UC: umbilical cord; BM: bone marrow; AT: adipose tissue; IV: intravenous; SLEDAI: Systemic Lupus Erythematosus Disease Activity Index; BILAG: The British Isles Lupus Assessment Group; SRI: SLE Responder Index; GARP: glycoprotein-A repetitions predominant.

## Data Availability

No new data were created or analyzed in this study. Data sharing is not applicable to this article.
